# Genetic Analysis and Status of Brown Bear Sub-Populations in Three National Parks of Greece Functioning as Strongholds for the Species’ Conservation

**DOI:** 10.3390/genes13081388

**Published:** 2022-08-04

**Authors:** Tzoulia-Maria Tsalazidou-Founta, Evangelia A. Stasi, Maria Samara, Yorgos Mertzanis, Maria Papathanassiou, Pantelis G. Bagos, Spyros Psaroudas, Vasiliki Spyrou, Yorgos Lazarou, Athanasios Tragos, Yannis Tsaknakis, Elpida Grigoriadou, Athanasios Korakis, Maria Satra, Charalambos Billinis, ARCPROM project

**Affiliations:** 1 Faculty of Veterinary Medicine, University of Thessaly, 43100 Karditsa, Greece; 2 Department of Computer Science and Biomedical Informatics, University of Thessaly, 35100 Lamia, Greece; 3 Department of Pathology, Faculty of Medicine, University of Thessaly, 41100 Larissa, Greece; 4 Callisto Wildlife and Nature Conservation Society, 54621 Thessaloniki, Greece; 5 Faculty of Animal Science, University of Thessaly, 41222 Larissa, Greece; 6 The Rodopi Mountain-Range National Park (RMNP), Mesochori Paranestiou, 66035 Paranesti, Greece; 7 Northern Pindos National Park Management Agency Aspraggeloi PC 44007, Municipality of Zagori, 45221 Ioannina, Greece; 8 Faculty of Public and One Health, University of Thessaly, 43100 Karditsa, Greece

**Keywords:** *Ursus arctos*, microsatellite loci, genetics, conservation, population structure

## Abstract

In order to optimize the appropriate conservation actions for the brown bear (*Ursus arctos* L.) population in Greece, we estimated the census (Nc) and effective (Ne) population size as well as the genetic status of brown bear sub-populations in three National Parks (NP): Prespa (MBPNP), Pindos (PINDNP), and Rhodopi (RMNP). The Prespa and Pindos sub-populations are located in western Greece and the Rhodopi population is located in eastern Greece. We extracted DNA from 472 hair samples and amplified through PCR 10 microsatellite loci. In total, 257 of 472 samples (54.5%) were genotyped for 6–10 microsatellite loci. Genetic analysis revealed that the Ne was 35, 118, and 61 individuals in MBPNP, PINDNP, and RMNP, respectively, while high levels of inbreeding were found in Prespa and Rhodopi but not in Pindos. Moreover, analysis of genetic structure showed that the Pindos population is genetically distinct, whereas Prespa and Rhodopi show mutual overlaps. Finally, we found a notable gene flow from Prespa to Rhodopi (10.19%) and from Rhodopi to Prespa (14.96%). Therefore, targeted actions for the conservation of the bears that live in the abovementioned areas must be undertaken, in order to ensure the species’ viability and to preserve the corridors that allow connectivity between the bear sub-populations in Greece.

## 1. Introduction

The brown bear (*Ursus arctos,* L.) is one of the most widespread large carnivores with a distribution covering [1] several continents (Europe, Asia, and North America) [2,3]. Specifically, in Europe, the brown bear’s range is fragmented into ten populations found across large geographical regions [1,4], while the lowest-latitude fringe of their European range is found in Greece, covering circa 25,000 km^2^ of suitable habitat. Animal populations living at the edge of their range, such as brown bears in the Hellenic peninsula, are important biological units as they can provide critical information on the evolutionary processes involved in the adaptation to local environments [1,5]. In Greece brown bears are a solitary and cryptic species, which are observed at relatively low densities most of the time and whose territory and distribution covers very large areas [6]. Specifically, brown bears in Greece survive in two previously totally disconnected populations: in Pindos and Peristeri, which are adjacent mountain ranges in western Greece, and in the Rhodopi mountain range in the eastern part of the mainland, both connected to the Dinaric-Pindos and East Balkan bear populations, respectively. The Pindos/Peristeri bear population comprises two geographical sub-nuclei populations: one is located in the Peristeri range, including Prespa NP, and the other in the main mountain range of Pindos (North Pindos and Central–Southern Pindos [4]). They are all connected to the bear populations of Albania and North Macedonia, while the Rhodopi population is connected to the East Balkan population in Bulgaria [7,8,9]. A variety of management and protection approaches has been suggested, in the context of a comprehensive human–bear conflict management plan, including measures relating to local communities’ property protection and compensation, habitat conservation, and bear population control [10,11]. In a decision-making process regarding the appropriate orientation and adjustment of conservation actions for the brown bears in Greece, one must consider the demographic dynamics and their specificities characterizing the targeted brown bear sub-populations. Towards this direction acquisition of genetic information, that reveals a range of attributes regarding brown bear population current status and subsequently the species conservation status needs and perspectives, is of outstanding importance and significance for the long-term survival of the species in the abovementioned mountainous regions of Greece.

Over the last two decades, non-invasive genetic sampling (NIGS) has become available for the demographic study of species [12,13,14,15], and has emerged as a reliable and cost-efficient tool to deal with the inherent difficulties of studying large carnivores [3,16]. NIGS does not require live trapping and individual marking since other collecting methods, e.g., hair traps, for subsequent genetic analysis are successfully used [17,18,19]. Moreover, even though the DNA provided from non-invasive samples sometimes has poor quality and quantity, it could be successfully used for genotyping variable molecular markers, such as microsatellite loci [15,20,21,22,23,24].

Based on the above, in this study we focused on the genetic analysis of 10 microsatellite loci using DNA received from hair samples of brown bears living in three regions in Greece: Prespa National Park (MBPNP), Pindos National Park (PINDNP), and Rhodopi National Park (RMNP). The aim of the study was to obtain the following information:

(a) The population structure (number of bears present in the three sub-populations, sex ratio, etc.) through the estimation of the census (Nc) and effective (Ne) population size and testing for signatures of past bottlenecks.
(b) The genetic variability of the brown bears in Greece and possible differentiation between the eastern and the two western sub-populations,
(c) Evidence of possible connectivity and migration of bears between the abovementioned study areas.

The results of the research will indicate the viability status of the surveyed populations and are expected to have a direct impact on the species’ conservation and management policies and actions in the studied areas. Moreover, these data are crucial parameters that will lead to a mid- and long-term conservation planning and will help to establish a balanced coexistence between humans and brown bears in the Prespa, Pindos, and Rhodopi national parks.

## 2. Materials and Methods

### 2.1. Sampling

The samples were collected from 2020 till 2021 from a network of 569 hair traps in total installed in the areas of Prespa (MBPNP) (51), Pindos (PINDNP) (262), and Rhodopi (RMNP) (256) national parks in the frame of the LIFE “ARCPROM” project (LIFE18NAT/GR/00768), with implementation from 2019–2024. Non-invasive genetic sampling was based on the ubiquitous marking and rubbing behaviour of bears on wooden poles of the telephone and electricity network (power poles) in Greece [25,26]. The hair traps (coiled barbed wire) were placed on selected power poles according to the previously detected bear rubbing activity (signs of bites, scratches, etc.) in the surveyed areas. Power poles are made of wood (approximately 30–50 cm in diameter and 10 m high) and processed with a wood preservative (i.e., coal tar creosote, a substance derived from tars) to resist decay and insect damage. Depending on topography, poles are usually placed 50 to 100 m apart, and vegetation is cleared 5 m from each side of the power pole line [3]. After collection, hair samples were placed in paper envelopes containing silica gel desiccant and stored at −20 °C. In total, 472 samples were collected and used in this project: 96 samples from MBPNP, and 170 samples from PINDNP, and 206 samples from RMNP.

### 2.2. DNA Extraction

Each tuft of hair on a set of barbs was considered a sample. Hair samples were collected without contact to human skin; they were placed in uniquely numbered paper envelopes labelled with the exact location, coordinates, date, and height of collection and then stored at −20 °C in Ziplock bags with silica gel until being analyzed. For all the collected samples, DNA extraction was performed using the DNA Mini kit (QIAGEN, Hilden, Germany) following the manufacturer’s instructions.

### 2.3. PCR Amplification

**Figure 1:**
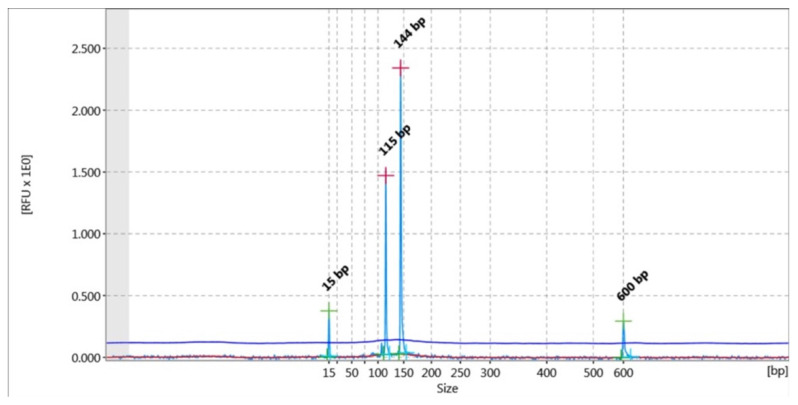
An XY brown bear: 2 bands appeared post capillary electrophoresis via QIAxcel (144 bp and 115 bp). The 15 bp and 600 bp peaks were derived from the QX DNA Size Marker that was used (see Supplementary Material).

DNA was amplified for 10 commonly used microsatellite loci: G10H, Mu26, G1D, G10X, G1A, G10P, G10C, Mu59, G10L, Mu50, as well as ZFX and the SRY gene [27,28,29]. Gender identification was performed using specific primers that co-amplify a bear-specific Y marker (SRY gene) and a bear-specific X marker (ZFX gene), according to Pages et al. (2009) [30]. If the sample is a male bear, 2 bands (144 bp and 115 bp) appear post capillary electrophoresis via QIAxcel (Figure 1) and 1 band (144 bp) appears in the case of a female bear. The primers that were used for each locus are shown in Table 1.

Given that co-amplification reduced the success rates for hair samples, the microsatellite loci were not coamplified, but we performed separate PCR assays for each microsatellite locus in a 15 μL total volume reaction using 7.5 μL HotStarTaq Master Mix (contains 0.1 units/μL HotStarTaq DNA Polymerase, PCR Buffer with 3 mM MgCl_2_, and 400 µM of each dNTP), 3 μL DNA, 1 p/μL for each primer and RNase-Free Water. The amplification conditions used were denaturation at 94 °C for 5 min, 40 cycles at 94 °C for 30 s, primer hybridization at 58 °C for 45 s, and elongation at 72 °C for 1 min. The final elongation was performed at 72 °C for 8 min. Thermal cycling was performed using an MJ Research (Peltier Thermal Cycler) PTC-200 thermocycler with 96-well “gold” blocks. Because of the low quantity of DNA, the samples were not electrophorized after the DNA extraction process.

To evaluate the amplification of the specific locus, all PCR products were electrophorized using 2% agarose gel. In order to identify the exact length of each microsatellite locus that was successfully amplified, we performed capillary electrophoresis through the QIAxcel Advanced System.

### 2.4. Capillary Electrophoresis

High-resolution capillary electrophoresis was performed using a QIAxcel DNA high-resolution kit (Qiagen, Hilden, Germany) on a QIAxcel Advanced System (Qiagen, Hilden, Germany), according to the manufacturer’s instructions. To our knowledge it is the first time that QIAxcel is used for microsatellite analysis in brown bears (see Supplementary Materials and Methods and Figure S1).

In order to increase the success rate and the accuracy of the procedure we performed separate PCR assays for each microsatellite locus and did not co-amplify the loci. The identification of the exact length was conducted through capillary electrophoresis, after each successful PCR. To minimize the genotyping errors in the final data set, PCR assays were performed twice for all samples, and each assay always included a negative control (blank). Subsequently, capillary electrophoresis was performed for each successful PCR product, only when the negative control (blank) was clear (absence of DNA product). Some samples were identified as mixed samples (samples with hair from >1 bear) by evidence of ≥3 alleles at ≥1 locus [31]. In the final data set, low-quality and putatively mixed samples were excluded from further analyses [32]. Genotyping was repeated for a third time for (1) the loci of samples, which showed discrepancy between the first two PCR assays; (2) the loci of samples that were found homozygous, as each time a homozygous genotype is observed, there is the possibility that it represents a true heterozygote where consecutive dropout errors have occurred [33]; and (3) pairs of individuals that differed at only 1 or 2 loci. In each locus we accepted the alleles that were found at least twice for the heterozygous genotypes and three times for the homozygous genotypes. The DNA extracted from non-invasive sources (such as hair) is often at low concentrations and/or highly fragmented [29]. Therefore, due to the total volume of the DNA extracted, we could not perform more than three separate PCR assays in all loci for each sample. Finally, any complete genotype with missing information in four or more loci was characterized as unreliable and was excluded from all further analyses [26], while tests for allelic dropout and presence of null alleles were performed with Micro-Checker version 2.2.3 (Microsoft Corporation, Albuquerque, NM, USA) [34], and are analysed in Section 2.6.

### 2.5. DNA Fragment Analysis

**Figure 2:**
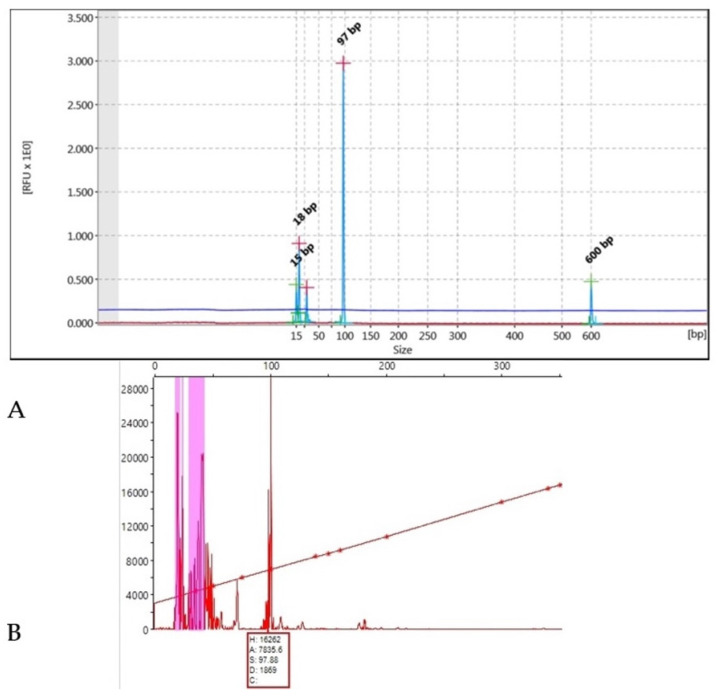
Homozygous sample for allele 97 bp of the G10C locus defined both by (**A**) the QIAxcel Advanced System and (**Β**) the ABI3500 genetic analyser.

The 10 microsatellite loci were also analysed using an ABI 3500 genetic analyser (Applied Biosystems^®^, Waltham, MA, USA), which allows the process of 1–5 different loci at the same time. This method was used in 10 samples to verify the size of the bands in each locus and to ascertain the accuracy of QIAxcel capillary electrophoresis (Figure 2). The main stages in this method are (1) PCR amplification with fluorescent dyes; (2) sample preparation with formamide and a marker; (3) capillary electrophoresis in the analyser; and (4) length analysis (Peak Scanner Software v.10, ThermoFischer Scientific, Thessaloniki, Greece).

### 2.6. Statistical and Computational Analysis

In order to identify possible siblings in the samples, Gimlet software was used [35]. Moreover, Cervus v3.0 (Kalinowski, Scotland)was used, in order to calculate various summary statistics for each locus, such as the number of individuals typed, the number of alleles, and the polymorphic information content (PIC) [36,37,38]. The identification of unique genotypes was performed through Dropout, while deviations from Hardy–Weinberg equilibrium (HWE) were calculated with Genepop v4.6 [39], with *p*-values coming from the Markov Chain method. Genepop was also used for the calculation of the observed and expected heterozygosity of each locus. Subsequently, BOTTLENECK was applied to compute, for each population and for each locus, the distribution of the heterozygosity expected from the observed number of alleles (k), given the sample size (*n*), under the assumption of mutation-drift equilibrium [40], and MICRO-CHECKER in order to test loci for allele dropout and errors made during the scoring of alleles with ‘stutter’ in our data [34,41].

NeEstimator v1.3. (Peel, Queensland, Australia) was used for estimating contemporary effective population size (Ne) using multi-locus diploid genotypes from population samples, using the linkage disequilibrium option [42]. In order to estimate Nc, the census population size, we used the Capwire R package [43], which is suitable for mark–recapture studies in small populations with non-invasive genetic sampling and single or multiple sampling sessions [44,45,46]. For such studies there are two main models: the equal capture model (ECM) which assumes that individuals have an equal probability of being captured [47,48,49,50], and the two-innate rates Model (TIRM) [47], under which the population is assumed to contain individuals that are easy to be captured and individuals that are not [43]. Since we use microsatellite loci, we decided to use TIRM as advised [43]. We obtained the maximum likelihood estimate (MLE) of the census population size along with the 95% confidence intervals using bootstrap with 1000 repetitions [47].

The structure of the population was studied using STRUCTURE 2.3.4 software (Pritchard, Oxford, UK), using a parameter set of a 50,000 burn-in period and 50,000 replications, combined with the Admixture Model (in which we let the software to infer the value for the α parameter of the Dirichlet distribution). The value of α was the same for all populations and the program uses a Uniform Prior [51]. Additionally, in order to assess the difference in the allele frequencies among different populations of a selected species, a measure known as Fst was calculated using Genepop v4.6 [39]. Finally, we used the BayesAss software [52], in order to estimate the recent migration rates between populations. BayesAss uses Bayesian inference with Markov chain Monte Carlo (MCMC). We used the default number of iterations (5,000,000) for the MCMC and we obtained the matrix of inferred (posterior mean) migration rates and the standard deviation of the marginal posterior distribution for each estimate.

## 3. Results

### 3.1. Samples That Were Analysed

An attempt was made to isolate DNA from all samples, followed by the application of PCR protocols for the 10 microsatellite loci as well as ZFX and SRY genes. Regarding hair samples, the quality and quantity of the hair roots determines the outcome of the microsatellite loci amplification [53]. In the present study, six or more genetic loci were successfully amplified in 257 samples in total: 59 of the 96 samples (61.5%) of Prespa; 77 of the 170 samples (45.3%) of Pindos; and in 121 of the 206 samples (58.7%) of Rhodopi. The analysis by MICRO-CHECKER revealed no indications of stuttering or large allele dropout.

### 3.2. Unique Genotypes and Sex Ratio

Based on their complete genotype for the 10 microsatellite loci, a total of 53, 65, and 77 unique individuals were identified in Prespa, Pindos, and Rhodopi NP, respectively. Moreover, regarding the sex ratio, in Prespa males were 2.5 times more numerous than the females (38 males/15 females); in Pindos males were 2 times more numerous than the females (44 males/21 females); and in Rhodopi males were 6 times more numerous than the females (67 males/11 females). The complete genotype and gender of all samples are shown in Supplementary Material Tables S1–S4.

### 3.3. Genetic Diversity

#### 3.3.1. Prespa NP

**Figure 3:**
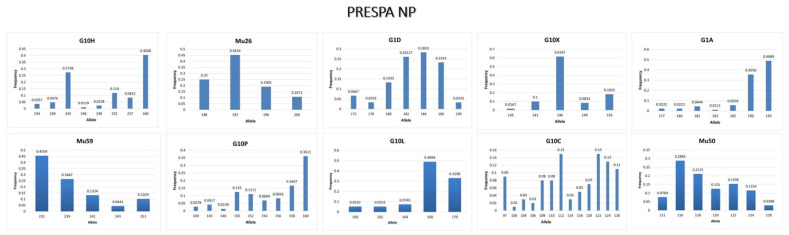
Allele frequency of each microsatellite locus of the brown bear population in Prespa NP.

Microsatellite data of the 10 microsatellite loci revealed abundant genetic diversity in the population from Prespa NP area. Table 2 shows the number of homozygotes and heterozygotes that are present at each locus of all samples. The number of alleles ranges from 4 (Mu26) to 13 (G10C) (Table 2), while the alleles with the highest frequency are shown in Figure 3.

The average observed heterozygosity (Ho) was 0.42 (range 0.09–0.83), and the average estimated heterozygosity (He) was 0.73 (range 0.57–0.90) for the 53 unique individuals, respectively (Table 3). The PIC [25] at each microsatellite locus was always higher than 0.5 (range 0.53 to 0.89), a threshold value considered to be highly informative for the evaluation of genetic variance [54,55] (Table 3).

We estimated that the 5 loci had significant deviations from the HWE (*p* < 0.001). In our analysis all loci seem to have a Fis value higher than 0.15 (excluding the loci G10X, G10L and Mu50). A Fis value higher than 0.15 indicates that high inbreeding levels occur in the population [56,57]. Moreover, G10H, Mu26, and G1D showed a P_ID-sib_ > 0.05, indicating a moderate presence of siblings and relatives.

#### 3.3.2. Pindos NP

**Figure 4:**
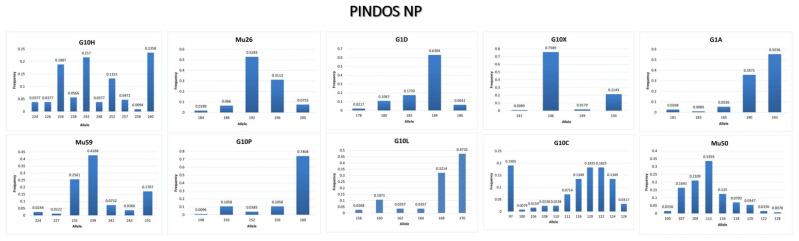
Allele frequency of each microsatellite locus of the brown bear population in Pindos NP.

Microsatellite data of the 10 microsatellite loci revealed abundant genetic diversity in the population of Pindos NP area. Table 4 shows the number of homozygotes and heterozygotes that are present at each locus of all samples. The number of alleles ranges from 5 (Mu26, G1D, G1A, G10P) to 11 (G10H) (Table 5), while the alleles with the highest frequency are shown in Figure 4. 

The average Ho was 0.6 (range 0.31–0.91), and the average He was 0.65 (range 0.38–0.86). The PIC [25] at each microsatellite locus was always higher (except from locus G10P and G10X) than 0.5 (range 0.32 to 0.84), a threshold value considered to be highly informative for the evaluation of genetic variance (Table 5). 

Seven loci had significant deviations from HWE (*p* < 0.001), as shown in Table 5. Regarding the Fis for the inbreeding existence [50], five loci seem to have an Fis value smaller than 0.15: Mu26, G10X, G1A, G10C, and Mu50. In Pindos NP, G10H, Mu26, G1D, and G10X showed a P_ID-sib_ > 0.05, indicating a moderate presence of siblings and relatives.

#### 3.3.3. Rhodopi NP

**Figure 5:**
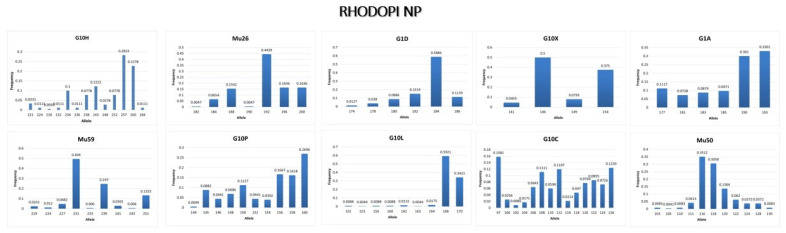
Allele frequency of each microsatellite locus of the brown bear population in Rhodopi NP.

Microsatellite data of the 10 microsatellite loci revealed abundant genetic diversity in the population of Rhodopi NP. Table 6 shows the number of homozygotes and heterozygotes that are present at each locus of all samples. The number of alleles ranges from 4 (G10X) to 13 (G10C) (Table 6), while the alleles with the highest frequency are shown in Figure 5.

Τhe average Ho was 0.54 (range 0.13–0.86), and the average He was 0.72 (range 0.52–0.90). The PIC [25] at each microsatellite locus was higher than 0.5 (range 0.43 to 0.88), a threshold value considered to be highly informative for the evaluation of genetic variance. 

In Rhodopi seven loci, with the exception of G10X, Mu26, and Mu50, had deviations from HWE (*p* < 0.001) (Table 7). Regarding the Fis marker for the inbreeding existence [50], only two loci (G10L and Mu50) had a Fis value smaller than 0.15. The presence of loci with high Fis values suggests a considerable degree of inbreeding in this sub-population [56,57]. In Rhodopi NP, G10H, Mu26, and G1D showed a P_ID-sib_ > 0.05, indicating a moderate presence of siblings and relatives.

### 3.4. Bottleneck

**Figure 6:**
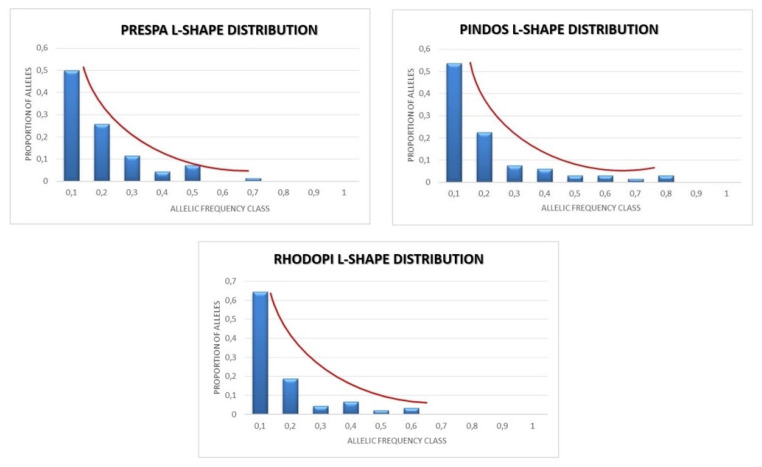
L-shaped distributions of the alleles’ frequencies for Prespa, Pindos, and Rhodopi NP.

We used the stepwise mutation model (SMM) [58], which is more suitable for microsatellite data [43], and our analysis showed that the population numbers have not recently decreased (*p*-value of the Wilcoxon test is equal to 0.72168, 0.99854, and 0.94727 for Prespa, Pindos, and Rhodopi NP, respectively). Therefore, in the three studied sub-populations, no evidence of a significant bottleneck was detected. The L-shaped distributions of the alleles’ frequencies, which are shown in Figure 6, corroborate the above.

### 3.5. Estimates of the Census Population Size (Nc) and Effective Population Size (Ne) in Prespa, Pindos, and Rhodopi NP

Regarding the population of Prespa, we found that Ne = 35 (95% confidence interval: 25–52) and that Nc = 191 (95% confidence interval: 150–222). Moreover, the mean arrest/sample ratio was 1.11 since 48 of the 53 samples were obtained once, four samples were obtained twice, and one sample was obtained three times. 

In Pindos NP, we found that Ne = 118 (95% confidence interval is from 66 to 371 individuals) and Nc = 202 (95% confidence interval: 175–300). The mean arrest/sample ratio was 1.18, since 56 of the 77 genotypes were obtained only from one sample. On the contrary, six of the 77 samples were obtained twice, and three samples were obtained three times.

Finally, in Rhodopi we found that Ne = 61 (95% confidence interval: 47–84) and Nc = 92 (95% confidence interval: 89–112). In Rhodopi, the mean arrest/sample ratio was 1.73 since 7 of the 121 samples were obtained once, 42 were obtained twice, 18 were obtained three times, 4 samples were obtained four times, and 7 samples were obtained seven times.

### 3.6. Inference of Population Genetic Structure

**Figure 7:**
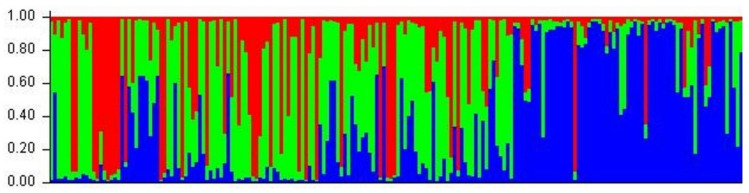
STRUCTURE results for the 3 sub-populations of brown bears: each individual is represented by a vertical line broken into 3 coloured segments, with lengths proportional to each of the 3 membership probabilities. With green we denote the Prespa population, with blue the Pindos population, and with red the Rhodopi population.

The basic results of genetic analysis of the three areas used in this study are summarized in Table 8. Additionally, we found that the mean Fst value is 0.0482 (between Prespa and Pindos), 0.0155 (between Prespa and Rhodopi), and 0.0696 (between Pindos and Rhodopi). An Fst value close to 0 shows a high breeding status between the populations. Moreover, the population structure bar plot generated by the STRUCTURE software shows the three populations with the estimated class membership probabilities. It is evident that the Pindos population is more genetically distinct, whereas Prespa and Rhodopi show mutual overlaps (Figure 7).

**Figure 8:**
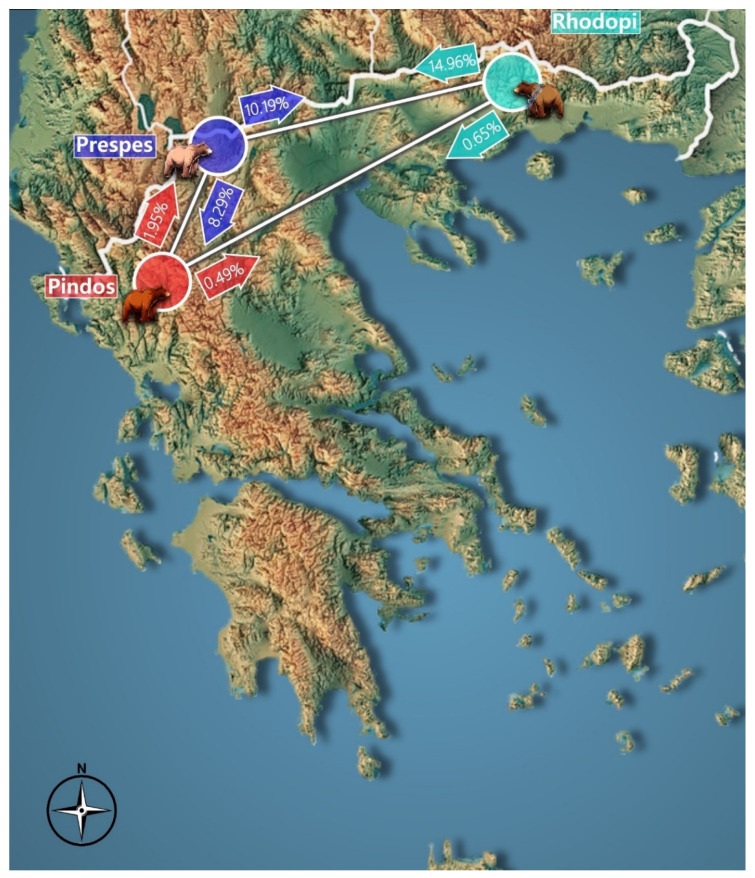
BAYESASS estimates migration rate between populations through gene flow.

To resolve these findings, we estimated the recent migration rate between populations and therefore the gene flow (Figure 8). The direction of the arrows denotes the estimated migration rates, from one population to another. The percentage denotes the fraction of the population into which individuals are migrating. 

## 4. Discussion

### 4.1. Conclusions from the Genetic Analysis in Prespa NP

To our knowledge, this is the first study that includes bears from the overall area of Prespa National Park (MBPNP), but there are studies that have conducted genetic analysis of bears from regions close or part of MBPNP (e.g., Peristeri, Kastoria, Amyntaio) [6,9,59]. Based on the genetic data produced from this study we found that in the Prespa population the microsatellite loci are informative for the estimation of the population size and we can use them for the analysis of genetic variance (PIC > 0.5). Regarding the genetic diversity, the census population size (Nc) and the effective population size (Ne), our findings agree with some previous studies [6,59]. Based on our genetic data retrieved from the 53 samples of this region, we estimated that the census population size (Nc) of the brown bear population of Prespa NP consists of 191 individuals, while Pylidis et al. found 109 individuals based on analysis of 30 samples received from Peristeri mountain, a mountain massif adjacent at the eastern border of Prespa lake basin [9].

As shown in Table 9, the inbreeding status of bears in Prespa shows high values of Fis (Fis > 0.15). Fis is the proportion of the variance in the sub-population contained in an individual (inbreeding coefficient) [50]. The low effective size of the population increases the impact of inbreeding and in the long term this may reduce the viability of Prespa population [60]. Moreover, the census population size of brown bears in Prespa is 5.45 times larger than the effective population size. These values were calculated using NeEstimator v1.3 with the option “linkage disequilibrium method”. The developers of the program [47] claim that accurate estimates are obtained with two or more captures/bear. Regarding Prespa the mean arrest/sample ratio was 1.11; therefore, this result should be treated with caution. Moreover, false-positive estimation of the population size can happen when the bears live in a larger area than the area of our study [48,49,50]. Therefore, population movements may affect positively the number of unique captures and negatively the number of re-captures [61]. Regarding the sex ratio, in Prespa we found that males were 2.5 times more than the females. This finding may be explained by the fact that “rubbing behaviour” on poles is more common in males than females [3,62].

### 4.2. Conclusions from the Genetic Analysis in Pindos NP

By using genetic data from hair samples collected exclusively from power poles, we obtained population estimates despite fairly low capture and recapture rates (in Pindos the mean arrest/sample ratio was 1.18). Based on these data we found that complete genotypes of the 10 microsatellite loci are informative (PIC > 0.5) and they show high genetic diversity of brown bears in Pindos NP.

Regarding the 65 unique bears found in Pindos, our findings show that they are characterized by relatively high genetic diversity and low values for Fis in the majority of loci. This declares a low inbreeding level of the population, which agrees with some of the previous studies [9,26] that are shown in Table 10. Therefore, high genetic diversity combined with low inbreeding coefficient and relatively high Nc/Ne ratio, shows that the bear population in Pindos NP is not currently threatened by genetic instability [26].

Specifically, our study (Table 10) estimated that the census population size (Nc) of brown bears in Pindos was 202 individuals while Pylidis et al. estimated that Nc = 299, conducting an analysis based on 97 unique samples [9]. In this sub-population, Nc is only 1.7 times larger than the effective population size and male bears were two times the number of females. Overall, at this moment Pindos is a population that seems to be able to maintain its genetic diversity and hopefully future studies will confirm this finding.

### 4.3. Conclusions from the Genetic Analysis in Rhodopi NP

Non-invasive genetics of the brown bear sub-populations of Rhodopi have been conducted in the past [4,9]. However, our study is the first in the area which includes an analysis based on a significantly high number of specimens, since 121 samples were genotyped in over than six microsatellite loci. Therefore, through our data the estimation of Nc and Ne of brown bears in Rhodopi may be more accurate. 

Table 11 compares our data with findings from previous studies conducted in the same area, but with a smaller number of samples. Based on our genetic analysis the effective population Ne = 61 (95% confidence interval: 47–84), while the census population in this area is expected to consist of 92 individuals, which agrees with the estimation by Pylidis et al. [9]. Moreover, the genetic diversity of 77 unique bear samples analysed in our study is relatively high (PIC > 0.5). 

Regarding the inbreeding values, a high Fis ( >0.15) implies a considerable degree of inbreeding [56,57], which was found in Rhodopi. Knowledge of the relative magnitudes of Ne and Nc, as expressed by the ratios Ne/Nc, are important for disentangling the relative risks that demographic, environmental, and genetic factors might pose for population persistence [63]. Moreover, the census population size of brown bears in Rhodopi is only 1.51 times larger than the effective population size. Expressed simply, a population with a high Nc relative to Ne will lose genetic diversity more quickly than an equal-sized population with a lower Nc/Ne ratio [64]. In Rhodopi, the mean arrest/sample ratio was 1.73, and as stated earlier, the linkage disequilibrium method requires two or more captures/bear in order to be accurate. Moreover, false-positive estimation of the population size can happen when the bears live in a larger area than the area of non-invasive sampling [48,50].

In addition, only in the Rhodopi population and regarding sex ratio, we found a larger difference between the number of male and female bears, since the males were six times more numerous than the females. It is known that male bears are more likely to rub on trees and electricity poles, in order to mark their home range, especially during the breeding season, and this behaviour leads to a certain degree of uneven hair sampling between the two genders [3,62]. This fact could explain that males were 2–3 times more than the females, which we found in Prespa NP and Pindos NP. In order to elucidate this deviation, which may be either intrinsic or human related, we will have to conduct further studies in Rhodopi. Perhaps the combination of hair and another type of bear biological material (such as faeces) can overcome the sampling bias and the subsequent underestimation of the female bear numbers in similar studies.

### 4.4. Comparison of Genetic Data from the Three Project Areas

Non-invasive sampling has many advantages regarding the collection of genetic material from large carnivores [65]. On the contrary, invasive methods can be dangerous, time consuming, costly, and can injure and/or stress the animal. Among the disadvantages regarding the samples received non-invasively (usually hairs and faeces) is the small amount of genetic material that is selected and the probability of contamination by the environment. These factors make the amplification of genetic markers through PCR difficult, while specific genetic markers may be amplified more easily than others. Based on the aforementioned points, in some microsatellite loci non-invasive sampling leads to genotypic errors such as zero and/or false alleles [66]. In the present study, non-invasive genetic sampling was used in order to collect samples of brown bears that live in the areas of Prespa, Pindos, and Rhodopi NP. In total, 472 hair samples were collected and 257 samples (54.5%) were genotyped at 6–10 microsatellite loci. This is in accordance to the percentage of samples genotyped successfully in similar studies, which is around 50% [59,67,68].

The growing interest in microsatellite genotyping, combined with non-invasive genetic sampling, has led to the necessity of managing these data. Analysis of our genetic data showed high genetic diversity in all populations, high levels of inbreeding in Prespa and Rhodopi, and lower values of inbreeding in Pindos NP. Moreover, no sign of a significant bottleneck was detected in any of our sub-populations. Based on our genetic data, we showed that our three sub-populations can be successfully distinguished in two clusters with a clear distinction between the western (Pindos and Prespa NP) and eastern (Rhodopi) populations (Figure 7). Towards this direction are the findings of Pylidis et al. [9], who determined that the geographical populations of Peristeri (which is a part of the Prespa NP), Pindos, and Rhodopi host distinct genetic demes. 

Based on the Fst values, the population of Prespa seems to show a tendency of breeding more with the population of Rhodopi than with the population of Pindos, while the population of Rhodopi seems to be genetically distinguished from Pindos. Fst values close to 0 (or negative) mean that the examined populations have high levels of breeding and values > 0.05 indicate genetic isolation between populations, which means that the populations are not currently breeding with each other [69]. In the P_ID_SIB_ analysis for the three populations, the majority of loci under investigation showed moderate evidence for excessive presence of siblings or relatives in the sample [70,71]. Nevertheless, we did not proceed with individual analysis of siblings since this was out of scope in the particular study. As clearly shown in Figure 8, there is a notable gene flow from Rhodopi to Prespa (14.96%), and an interesting gene flow both from Prespa to Rhodopi (10.19%) and from Prespa to Pindos (8.29%). In brown bears, a long-range displacement of vagrant males (up to 360 km) has been recorded [72]. This could mean that the distance which separates Prespa from Rhodopi is well within the dispersal range capacity of the species, considering the established metapopulation in the Voras mountain massif located between Prespa and Rhodopi at the same latitude. Recent studies and data on bear occurrence in intermediate areas between Rhodopi and Prespa as well as the existing potential connectivity corridors confirm this assumption. On the other hand, the human-related landscape fragmentation and land use as well as natural topography features in Peristeri and Pindos mountain ranges may act as a barrier, limiting the dispersal intensity in both directions between sub-populations of Pindos and Prespa, as shown in Karamanlidis et al. [4]. Regarding natural topography features, we refer to high altitudinal variations, high mountain massifs and canyons as well as general topographic ruggedness forming topographic enclaves. Moreover, human-related landscape fragmentation can be directly related to transportation and other infrastructure development that disrupt bear habitats and sub-populations.

We conclude that the overall census population size and the total effective size of brown bears in the three studied areas is 485 and 214 individuals, respectively, which agrees with the findings of Pylidis et al. who found a total of Nc = 499 and Ne = 198 individuals in the same areas. However, we found an increased value of inbreeding in Prespa NP and Rhodopi NP, a high Nc/Ne ratio in Prespa, and a relative high male/female ratio in Rhodopi. These findings show that, at this moment, the sub-populations of Rhodopi and Prespa are more vulnerable compared to the population of Pindos. Therefore, the estimation and characterization of the population size in all the study areas should be continued on a long-term basis through the analysis of a larger number of specimens from the same regions. To ensure the viability of the species in these areas, it is necessary to take immediate and appropriately targeted actions for the conservation and management of these populations. This will provide safer answers regarding both population density and genetic variability, based on extended non-invasive sampling and monitoring programs. Finally, the above results also highlight the need to conserve the corridors that allow the connectivity of the bear sub-populations over large distances and geographic scales in the fragmented landscape context of Greece and the Balkans.

## Figures and Tables

**Table 1:** Primer sequence and length of the 10 microsatellite loci.

Primer Sequence		Length (bp)	References
**G10H F**	5′-CCCAACAAGAAGACCACTGTAA-3′	221–257	[28]
**G10H R**	5′-CCAGAGACCACCAAGTAGGATA-3′		
**G10L F**	5′-TGTACTGATTTAATTCACATTTCCC-3′	153–163	[28]
**G10L R**	5′-GAAGATACAGAAACCTACCCATGC-3′		
**Mu50 F**	5′-GTCTCTGTCATTTCCCCATC-3′	110–130	[27]
**Mu50 R**	5′-AACCTGGAACAAAAATTAACAC-3′		
**Mu26 F**	5′-GCCTCAAATGACAAGATTTC-3′	182–200	[20]
**Mu26 R**	5′-TCAATTAAAATAGGAAGCAGC-3′		
**G10P F**	5′-TACATAGGAGGAAGAAAGATGG-3′	145–159	[28]
**G10P R**	5′-AAAAGGCCTAAGCTACATCG-3′		
**Mu59 F**	5′-TGCTGCTTTGGGACATTGTAA-3′	219–251	[20]
**Mu59 R**	5′-CAATCAGGCATGGGGAAGAA-3′		
**G10C F**	5′-AAAGCAGAAGGCCTTGATTTCCTG-3′	97–116	[28]
**G10C R**	5′-GGGACATAAACACCGAGACAGC-3′		
**G1D F**	5′-ATCTGTGGGTTTATAGGTTACA-3′	172–184	[28]
**G1D R**	5′-CTACTCTTCCTACTCTTTAAGAG-3′		
**G10X F**	5′-CCCTGGTAACCACAAATCTCT-3′	132–154	[20,28]
**G10X R**	5′-TCAGTTATCTGTGAAATCAAAA-3′		
**G1A F**	5′-GACCCTGCATACTCTCCTCTGATG-3′	180–190	[28]
**G1A R**	5′-GCACTGTCCTGCGTAGAAGTGAC-3′		
**SRY-F**	5′ -TGGTCTCGTGATCAAAGGCGC-3′	115	[30]
**SRY-R**	5′-GCCATTTTTCGGCTTCCGTAAG-3′		
**ZF-F**	5′-GACAGCTGAACAAGGGTTG-3′	144	[30]
**ZF-R**	5′-GCTTCTCGCCGGTATGGATG-3′		

**Table 2:** Number of homozygotes, heterozygotes, and alleles that are present at each locus (Prespa NP).

Locus	Individuals	Heterozygotes	Homozygotes	Number of Alleles
**G10H**	42	13	29	8
**Mu26**	39	25	14	4
**G1D**	30	11	19	7
**G10X**	30	17	13	5
**G1A**	45	23	22	7
**G10P**	36	21	15	9
**G10C**	50	34	16	13
**Mu59**	34	5	29	5
**G10L**	47	32	15	5
**Mu50**	52	44	8	7

**Table 3:** Genetic information of the brown bear population from Prespa NP. Number of alleles (A); allele size per bp (R); expected heterozygosity (He); observed heterozygosity (Ho); *p*-value for Hardy–Weinberg Equilibrium (pHW); inbreeding coefficient (Fis); probabilities of identity (P_ID-sib_); frequency of null alleles (Fnull); polymorphic information content (PIC).

Locus	A	R (bp)	He	Ho	pHW	Fis	P_ID-sib_	Fnull	PIC
**G10H**	8	234–260	0.74	0.25	0.0000	0.5873	4.088 × 10^−1^	0.4320	0.699
**MU26**	4	188–200	0.69	0.47	0.0550	0.1430	1.820 × 10^−1^	0.0788	0.633
**G1D**	7	172–190	0.79	0.21	0.0000	0.5501	6.758 × 10^−2^	0.3696	0.763
**G10X**	5	136–154	0.57	0.32	0.0180	0.0209	3.528 × 10^−2^	−0.0118	0.529
**G1A**	7	177–193	0.63	0.43	0.0020	0.1975	1.717 × 10^−2^	0.1130	0.563
**G10P**	9	140–160	0.80	0.40	0.0038	0.2833	6.280 × 10^−3^	0.1559	0.777
**G10C**	13	97–126	0.90	0.64	0.0000	0.2501	1.930 × 10^−3^	0.1381	0.886
**MU59**	5	231–251	0.69	0.09	0.0000	0.7931	8.479 × 10^−4^	0.6598	0.646
**G10L**	5	160–170	0.64	0.60	0.0000	−0.0522	4.045 × 10^−4^	−0.0281	0.581
**MU50**	7	111–128	0.81	0.83	0.6495	−0.0315	1.451 × 10^−4^	−0.0210	0.788
**Mean**	7		0.73	0.42		0.28			0.69

**Table 4:** Number of homozygotes, heterozygotes, and alleles that are present at each locus (Pindos NP).

Locus	Individuals	Heterozygotes	Homozygotes	Number of Alleles
**G10H**	53	34	19	10
**Mu26**	53	40	13	5
**G1D**	23	9	14	5
**G10X**	56	25	31	4
**G1A**	56	34	22	5
**G10P**	52	16	36	5
**G10C**	63	57	6	11
**Mu59**	41	19	22	7
**G10L**	56	32	24	6
**Mu50**	64	58	6	9

**Table 5:** Genetic information about the population of brown bears from Pindos NP. Number of alleles (A); allele size per bp (R); observed heterozygosity (Ho); expected heterozygosity (He); *p*-value for Hardy–Weinberg Equilibrium (pHW); inbreeding coefficient (Fis); probabilities of identity (P_ID-sib_); frequency of null alleles (Fnull); polymorphic information content (PIC).

locus	A	R (bp)	He	Ho	pHW	Fis	P_ID-sib_	Fnull	PIC
**G10H**	10	234–260	0.84	0.64	0.0000	0.2402	3.447 × 10^−1^	0.1280	0.814
**MU26**	5	188–200	0.62	0.76	0.0000	−0.2210	1.710 × 10^−1^	−0.1286	0.552
**G1D**	5	172–190	0.57	0.39	0.0040	0.3161	9.081 × 10^−2^	0.2107	0.517
**G10X**	4	136–154	0.38	0.45	0.5072	−0.1732	6.096 × 10^−2^	−0.0939	0.324
**G1A**	5	177–193	0.57	0.61	0.0000	−0.0707	3.274 × 10^−2^	−0.0349	0.481
**G10P**	5	140–160	0.43	0.31	0.0000	0.2898	2.045 × 10^−2^	0.2260	0.401
**G10C**	11	97–126	0.86	0.91	0.0000	−0.0526	6.813 × 10^−3^	−0.0337	0.836
**MU59**	7	231–251	0.72	0.46	0.0001	0.3632	2.882 × 10^−3^	0.2086	0.673
**G10L**	6	160–170	0.66	0.57	0.0000	0.1404	1.338 × 10^−3^	0.0657	0.602
**MU50**	9	111–128	0.80	0.91	0.0439	−0.1371	4.976 × 10^−4^	−0.0758	0.764
**Mean**	6.7		0.65	0.6		0.13			0.6

**Table 6:** Number of homozygotes, heterozygotes, and alleles that are present at each locus (Rhodopi NP).

Locus	Individuals	Heterozygotes	Homozygotes	Number of Alleles
**G10H**	64	17	47	12
**Mu26**	72	45	27	7
**G1D**	61	8	53	7
**G10X**	33	16	17	4
**G1A**	64	39	25	6
**G10P**	63	40	23	10
**G10C**	74	51	23	13
**Mu59**	61	20	41	9
**G10L**	76	54	22	7
**Mu50**	78	67	11	9

**Table 7:** Genetic information about the population of brown bears from Rhodopi NP. Number of alleles (A); allele size per bp (R); observed heterozygosity (Ho); expected heterozygosity (He); *p*-value for Hardy–Weinberg Equilibrium (pHW); inbreeding coefficient (Fis); probabilities of identity (P_ID-sib_); frequency of null alleles (Fnull); polymorphic information content (PIC).

Locus	A	R (bp)	He	Ho	pHW	Fis	PID-sib	Fnull	PIC
**G10H**	12	221–266	0.82	0.27	0.0000	0.6791	3.554 × 10^−1^	0.5208	0.795
**MU26**	7	182–200	0.75	0.63	0.0505	0.1617	1.447 × 10^−1^	0.0845	0.700
**G1D**	7	172–186	0.63	0.13	0.0000	0.7917	7.010 × 10^−2^	0.6607	0.586
**G10X**	4	141–154	0.61	0.49	0.2855	0.2044	3.587 × 10^−2^	0.1079	0.516
**G1A**	6	177–193	0.77	0.61	0.0000	0.2112	1.399 × 10^−2^	0.1213	0.730
**G10P**	10	144–160	0.83	0.64	0.0000	0.2329	4.948 × 10^−3^	0.1286	0.798
**G10C**	13	97–126	0.90	0.69	0.0000	0.2339	1.531 × 10^−3^	0.1322	0.882
**MU59**	9	219–251	0.64	0.33	0.0000	0.4924	7.273 × 10^−4^	0.3375	0.592
**G10L**	7	152–170	0.52	0.71	0.0000	−0.3659	4.161 × 10^−4^	−0.1676	0.426
**MU50**	9	103–128	0.77	0.86	0.0439	−0.1205	1.629 × 10^−4^	−0.0691	0.728
**Mean**	8.4		0.72	0.54		0.3			0.68

**Table 8:** Average estimates of number of alleles (A), expected heterozygosity (He), observed heterozygosity (Ho), census population size (N_C_), effective population size (Ne), polymorphic information content (PIC), and inbreeding coefficient (Fis) in the three project areas.

Population	Number of Samples with >6 loci	Different Individuals	A	He	Ho	Nc	Ne	PIC	Fis
**Prespa**	59	53	7	0.73	0.42	191	35 (25–52)	0.69	0.28
**Pindos**	77	65	6.7	0.65	0.6	202	118 (66–271)	0.6	0.13
**Rhodopi**	121	77	8.4	0.72	0.54	92	61(47–84)	0.68	0.3

**Table 9:** Comparative table with studies carried out in Prespa NP and in areas close to Prespa (number of alleles (A); expected heterozygosity (He); observed heterozygosity (Ho); census population size (Nc); effective population size (Ne); inbreeding coefficient (Fis)).

Area of Population	Samples	A	He	Ho	Nc	Ne	Fis	Reference
**Prespa**	53	7	0.73	0.42	191	35	0.28	*This study*
**Kastoria**	82	5.8	0.548	0.584	219	39.5–49	0.07	*Tsaparis* et al., *2015* [59]
**Peristeri**	30	5.64	0.69	0.65	109	59.1	0.047	*Pylidis, 2021* [9]
**Amyntaio**	56	6.8	0.582	0.412	154	44	0.25	*Mertzanis* et al., *2018* [6] LIFE15NAT/GR/001108

**Table 10:** Comparative table with studies carried out in Pindos (number of alleles (A); expected heterozygosity (He); observed heterozygosity (Ho); census population size (N_C_); effective population size (Ne); inbreeding coefficient (Fis)).

Area of Population	Samples	A	He	Ho	Nc	Ne	Fis	Reference
**Pindos**	65	6.7	0.65	0.6	202	118	0.13	*This study*
**Pindos**	159	6.42	-	0.70	-	182.3	-	*Karamanlidis, 2015* [26]
**Pindos**	47	5–8	0.76	0.77	76		-	*Karamanlidis, 2010* [2]
**North Pindos**	65	5.47	0.658	0.67	-	65–149.8	-	*Karamanlidis, 2018* [4]
**South-Central Pindos**	99	5.76	0.680	0.68	-	80.5–148.7	-	*Karamanlidis, 2018* [4]
**Pindos**	97	5.27	0.64	0.61	299	97.4	0.042	*Pylidis, 2021* [9]

**Table 11:** Comparative table with studies carried out in Rhodopi (number of alleles (A); expected heterozygosity (He); observed heterozygosity (Ho); census population size (N_C_); effective population size (Ne); inbreeding coefficient (Fis)).

Area of Population	Samples	A	He	Ho	Nc	Ne	Fis	Reference
**Rhodopi**	77	8.4	0.72	0.54	92	61	0.3	*This study*
**Rhodopi**	22	6.09	0.73	0.71	91	42.2	0.021	*Pylidis, 2021* [9]
**Rhodopi**	15	6.529	0.745	0.808	-	-	-	*Karamanlidis, 2018* [4]

## Data Availability

Data of this study are presented within the manuscript and the Supplementary Materials.

## Supplementary Materials

The following supporting information can be downloaded at: https://www.mdpi.com/article/10.3390/genes13081388/s1 Supplementary Materials and Methods: Capillary electrophoresis. Figure S1: Photos of the digital gel images and electropherograms via QIAxcel. Table S1: Complete genotype and gender of the 53 unique brown bears of Prespa NP, Table S2: Complete genotype and gender of the 65 unique brown bears of Pindos NP, Table S3: Complete genotype and gender of the 77 unique brown bears of Rhodopi NP (*n* = 1–60), Table S4: Complete genotype and gender of the 77 unique brown bears of Rhodopi NP (*n* = 61–77).
