# Supplementary material for: Genetic Analysis and Status of Brown Bear Sub-Populations in Three National Parks of Greece Functioning as Strongholds for the Species’ Conservation
Source: Genes (Basel). 2022 Aug 4;13(8):1388. doi: 10.3390/genes13081388 (PMC9407276; doi:10.3390/genes13081388)
Supplement: Supplementary file 1 [file genes-13-01388-s001.zip › genes-1713389-supplementary.pdf]

Supplementary materials and methods

Capillary electrophoresis

A QX DNA Size Marker (Qiagen) with 10 fragment sizes ranging in size from 25 to 500 bp was used to identify the PCR products' size. A QX Alignment Marker (Qiagen), which consisted of 15-600 bp fragments, was injected into the cartridge with each sample.

The 0M800 method in the ScreenGel® software (Qiagen) was used for all analyses; this corresponds to a 10sec sample injection time at 5 kV and 800sec separation time at 3 kV. The QIAxcel system injected 0.1 µl of 20 µl PCR products into a cartridge for analysis. The retention time of the PCR fragments relative to the 15-bp and 600-bp QX Alignment Marker fragments was calculated using the ScreenGel software (Qiagen). The PCR product sizes were then determined by comparing the retention time with the QX DNA Size Marker. The ScreenGel software produces a digital gel image and an electropherogram for fragment analysis (Figure 1). (Indicative photos of the digital gel images and electropherograms of PCR products are shown in supplementary material – Figure S1)

The high detection sensitivity provided by the QIAxcel Advanced System enables robust results even with low concentrations of nucleic acid. QIAxcel Advanced system has a high resolution for fragments smaller than 0.5 kb and ensures high accuracy and confidence in data interpretation. Sample consumption is less than 0.1 µl per analysis, saving precious samples for further downstream analysis.

Figure S1: Photos of the digital gel images and electropherograms via Qiaxcel

G10H: allele 238bp

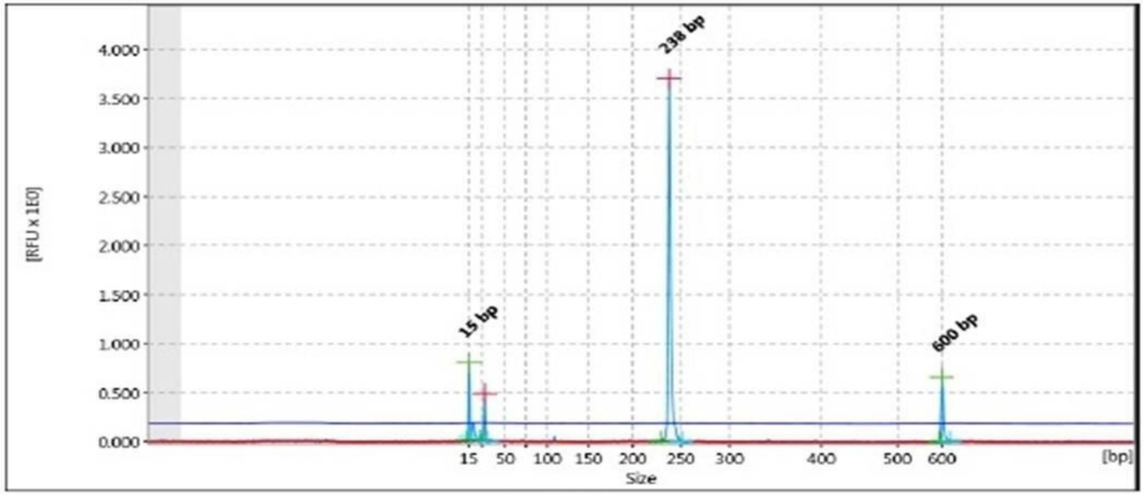

Figure: 149

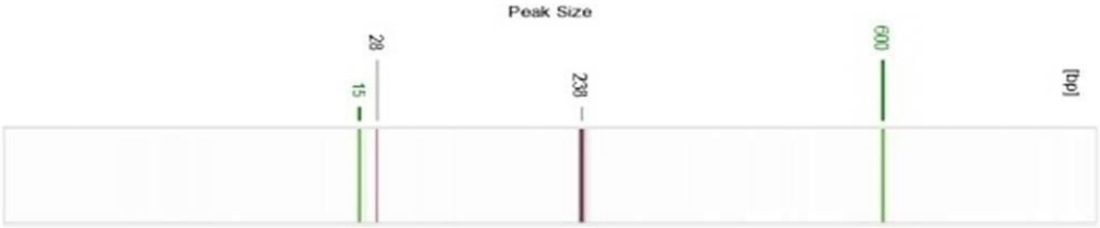

G1D: allele 184bp

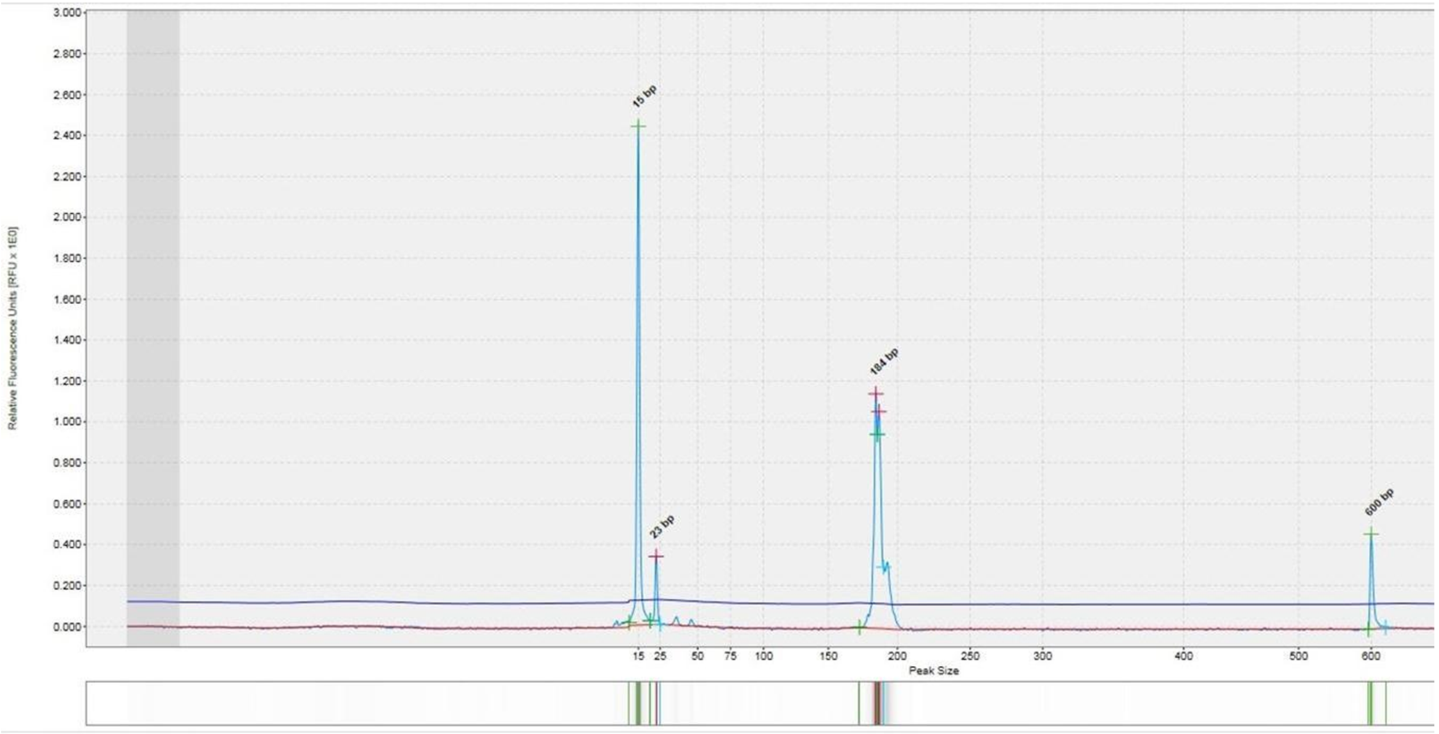

G10C: 97bp

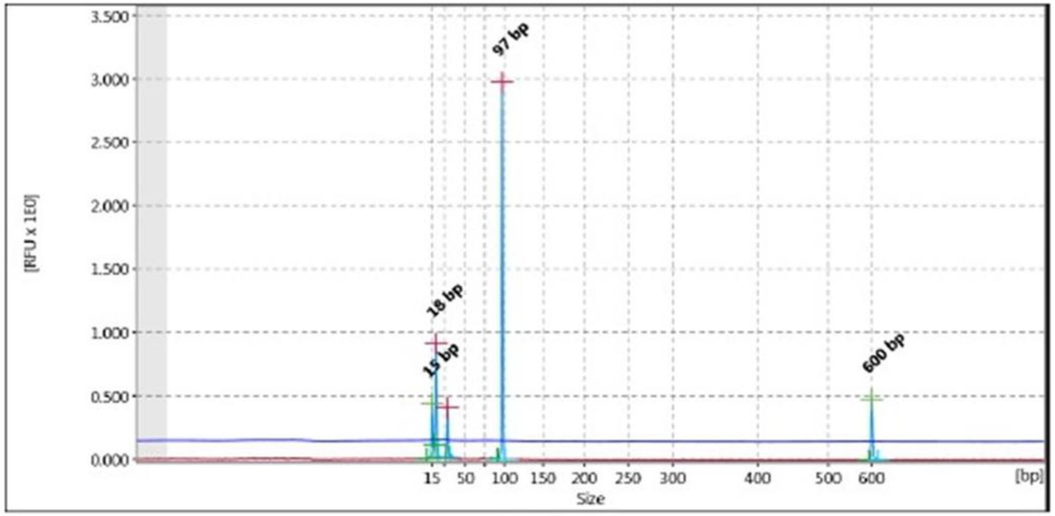

Figure: 35

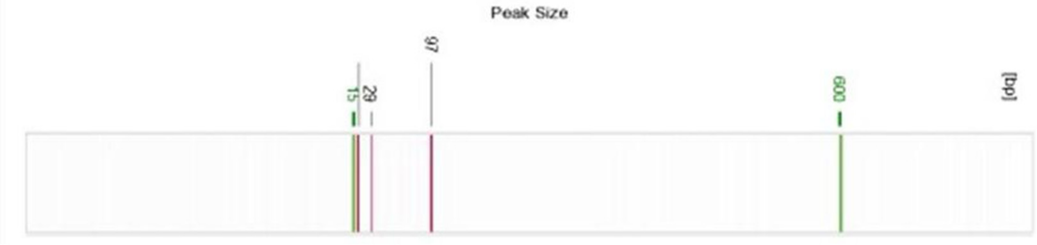

Mu26: 188 – 192 bp

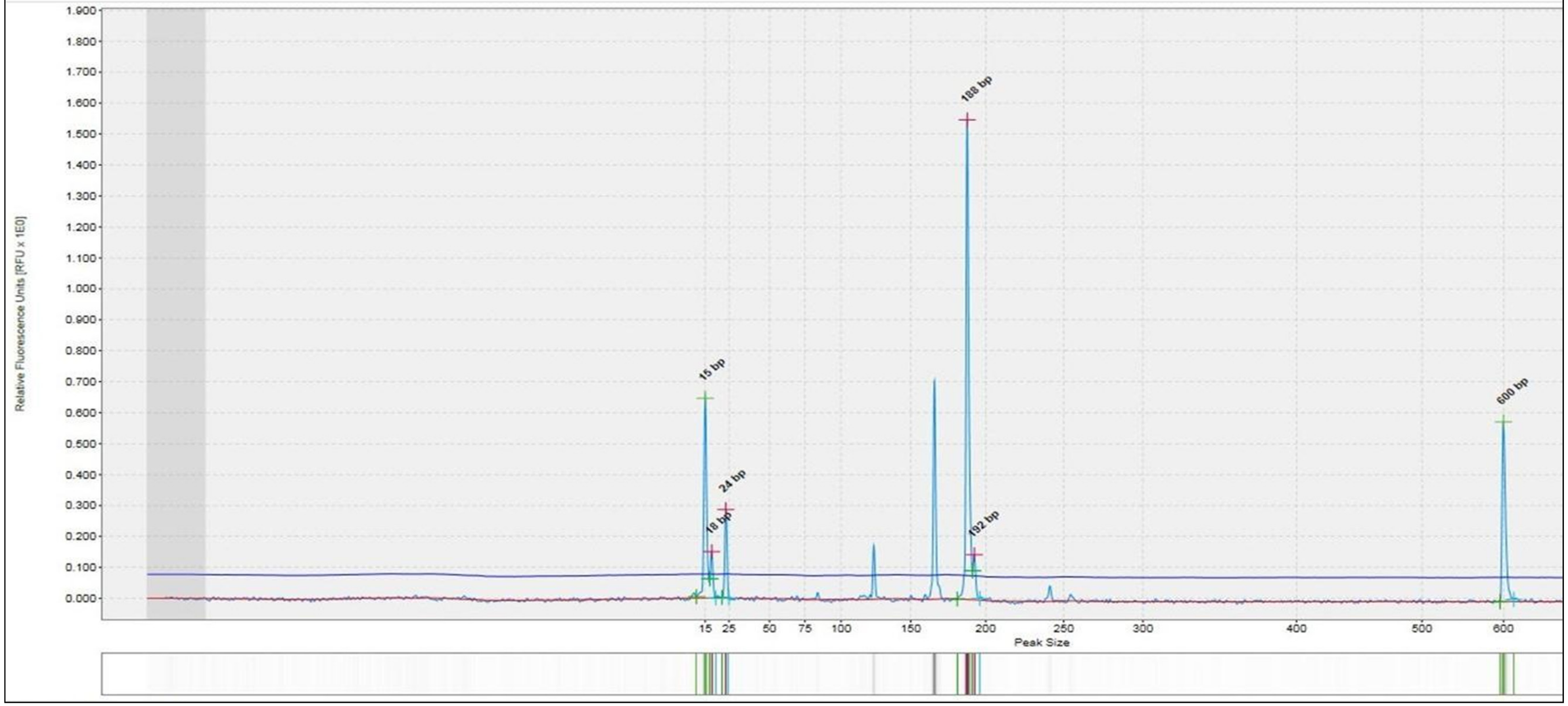

Mu59:239bp

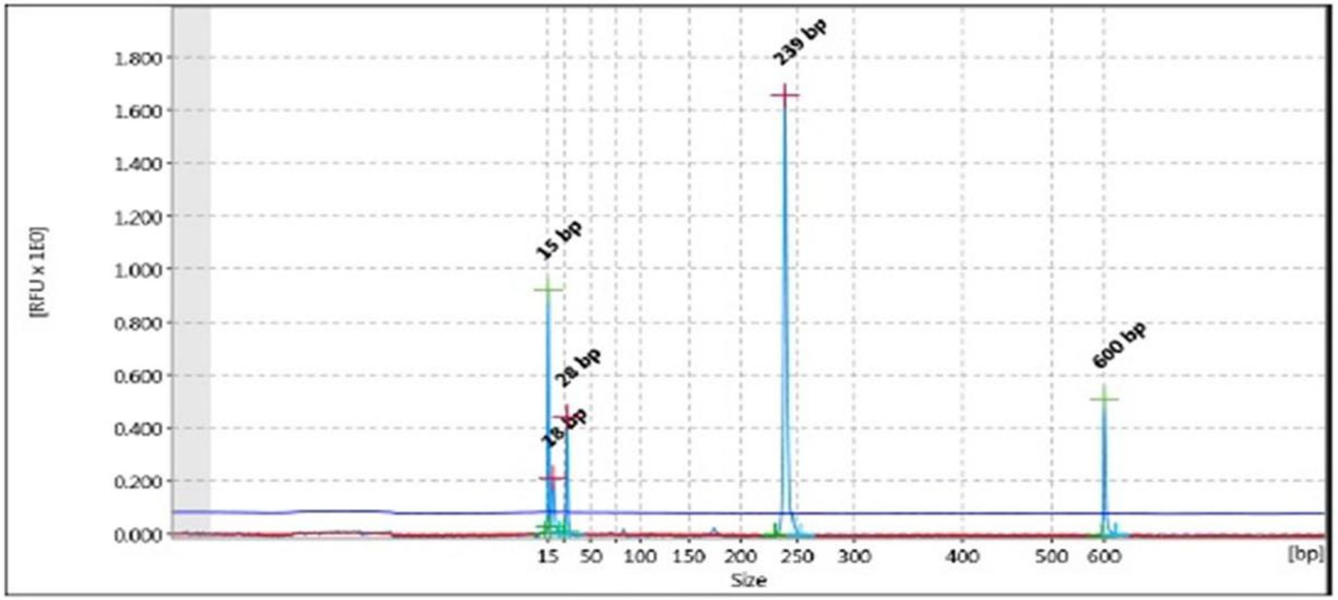

Figure: 105

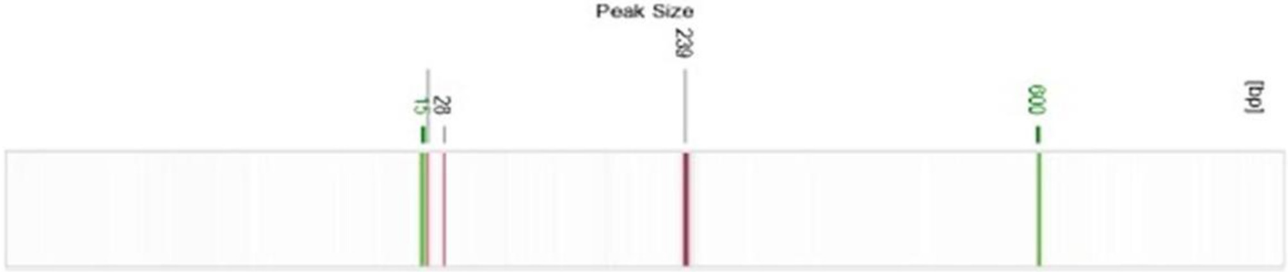

Mu50:111bp

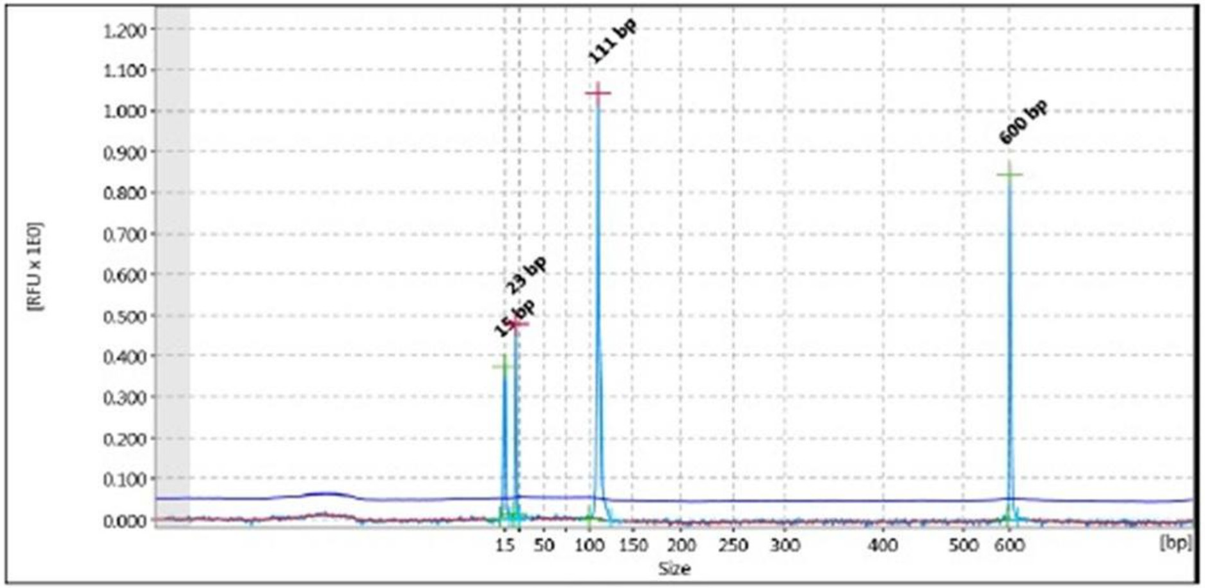

Figure: 171

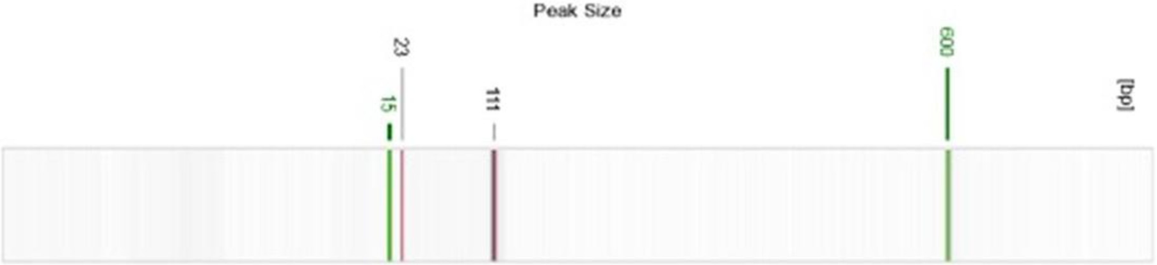

Table S1: Complete genotype and gender of the 53 unique brown bears of Prespa NP

A/A	Lab code	Gender	G10H (221-257bp)		Mu26 (182-200bp)		G1D (172-184bp)		G10X (132-154bp)		G1A (180-190bp)		G10P (145-160bp)		G10C (97-126bp)		MU59 (219-251bp)		G10L (153-163bp)		MU50 (110-130bp)	
1	UR25-XY	XY	260	260	192	196	0	0	146	154	193	193	152	160	114	126	241	251	160	164	122	122
2	UR26-XY	XY	243	243	192	192	184	184	0	0	190	193	154	160	126	126	239	239	168	170	118	118
3	UR27-XY	XY	243	243	192	192	0	0	146	149	193	193	154	160	126	126	239	239	168	170	118	118
4	UR28-XY	XY	243	243	192	192	184	184	146	149	193	193	152	158	124	126	243	243	168	170	122	122
5	UR29-XY	XY	260	260	192	192	182	182	146	149	185	190	145	150	110	110	241	241	168	170	116	122
6	UR30-XY	XY	260	260	192	196	186	186	146	146	193	193	158	158	112	120	241	251	162	164	122	124
7	UR32-XY	XY	260	260	0	0	0	0	0	0	193	193	156	156	112	112	0	0	168	170	116	118
8	UR33-XY	XY	243	246	192	192	182	182	146	149	193	193	152	160	124	124	231	231	168	170	118	120
9	UR198-XX	XX	243	252	0	0	180	184	0	0	193	193	0	0	108	112	0	0	168	168	111	118
10	UR199-XY	XY	243	252	188	196	0	0	0	0	190	193	0	0	108	120	0	0	168	168	120	124
11	UR200-XY	XY	0	0	200	200	186	186	0	0	190	193	156	160	110	120	239	251	160	164	120	128
12	UR202-XY	XY	0	0	0	0	0	0	141	141	185	190	160	160	120	124	0	0	168	168	111	120
13	UR204-XX	XX	260	260	188	192	0	0	0	0	190	190	160	160	0	0	0	0	168	168	111	116
14	UR205-XX	XX	260	260	188	192	0	0	0	0	190	190	0	0	110	114	0	0	164	168	111	116
15	UR11-XY	XY	260	260	192	192	182	182	146	146	193	193	150	152	124	126	239	239	168	170	116	120
16	UR12-XY	XY	243	252	192	196	186	186	141	154	181	190	150	156	97	97	251	251	168	168	116	118
17	UR13-XY	XY	257	257	192	196	0	0	146	146	0	0	150	158	118	120	239	239	160	164	116	116
18	UR14-XX	XX	243	243	200	200	0	0	146	149	183	190	148	154	97	104	231	231	0	0	118	120
19	UR17-XX	XX	234	234	196	200	186	186	0	0	181	185	145	145	97	104	241	241	168	168	118	124
20	UR18-XY	XY	260	260	0	0	0	0	141	141	193	193	152	158	122	124	231	231	0	0	0	0
21	UR19-XY	XY	0	0	192	192	0	0	0	0	190	193	160	160	112	118	0	0	168	170	116	118
22	UR20-XY	XY	243	243	192	192	0	0	0	0	177	190	152	160	112	112	231	231	168	170	118	122
23	UR22-XY	XY	260	260	188	188	182	182	146	146	190	193	152	160	124	126	239	239	168	170	118	118
24	UR24-XY	XY	0	0	192	192	0	0	0	0	180	180	154	160	122	126	0	0	168	170	111	116
25	UR31-XY	XY	260	260	196	196	0	0	146	154	190	190	158	158	112	122	241	241	162	164	122	124
26	UR182-XX	XX	0	0	0	0	0	0	0	0	190	190	154	156	112	122	231	231	168	170	111	122
27	UR184-XX	XX	0	0	188	200	180	182	0	0	190	190	0	0	122	122	0	0	168	170	116	124
28	UR185-XX	XX	260	260	0	0	186	186	0	0	0	0	160	160	108	108	0	0	168	170	116	118
29	UR187-XY	XY	0	0	188	196	172	184	146	154	0	0	0	0	106	110	0	0	168	170	120	124
30	UR188-XX	XX	243	243	196	200	190	190	0	0	193	193	150	160	112	120	231	231	170	170	118	124
31	UR189-XX	XX	260	260	0	0	0	0	0	0	0	0	158	158	108	110	231	231	168	170	116	116
32	UR190-XX	XX	257	257	0	0	0	0	0	0	190	193	140	140	106	126	0	0	168	170	111	116
33	UR192-XX	XX	260	260	192	192	0	0	0	0	193	193	0	0	0	0	231	231	168	168	111	116
34	UR194-XY	XY	260	260	192	192	172	172	0	0	0	0	0	0	110	110	231	231	168	170	116	118
35	UR195-XY	XY	0	0	0	0	180	184	0	0	190	190	0	0	97	97	231	231	168	170	116	116
36	UR242-XY	XY	243	243	188	188	0	0	146	146	190	193	158	160	122	122	239	239	168	170	118	122
37	UR243-XY	XY	238	252	188	196	178	180	146	146	181	185	0	0	97	104	0	0	0	0	116	120
38	UR244-XY	XY	260	260	188	196	186	186	146	146	193	193	152	160	122	124	231	239	170	170	120	124
39	UR245-XY	XY	252	257	188	196	172	182	0	0	193	193	0	0	114	122	0	0	0	0	116	120
40	UR246-XY	XY	252	252	188	192	182	184	136	146	0	0	0	0	100	108	0	0	168	168	116	120
41	UR248-XY	XY	260	260	188	188	0	0	146	154	190	193	0	0	108	124	239	239	168	170	116	122
42	UR252-XY	XY	243	252	188	192	182	182	146	146	190	193	160	160	122	126	0	0	0	0	122	128
43	UR253-XY	XY	260	260	188	200	184	184	146	146	190	193	156	158	112	120	241	243	170	170	122	128
44	UR258-XY	XY	243	252	188	196	186	186	146	146	190	193	150	150	108	118	0	0	162	164	116	124
45	UR259-XY	XY	243	243	0	0	180	184	0	0	0	0	0	0	112	112	231	231	168	168	116	118
46	UR260-XY	XY	0	0	188	192	180	184	146	154	193	193	158	160	122	124	239	239	160	162	116	124
47	UR262-XY	XY	234	257	188	192	180	184	146	154	193	193	0	0	122	124	239	239	160	162	118	124
48	UR263-XX	XX	0	0	192	192	178	180	0	0	185	193	0	0	124	124	231	231	168	168	116	120
49	UR264-XX	XX	238	248	192	200	184	184	146	154	190	193	160	160	118	122	231	231	170	170	120	122
50	UR268-XX	XX	238	248	188	196	184	184	146	154	177	190	160	160	118	122	231	231	0	0	122	124
51	UR270-XY	XY	0	0	188	192	0	0	146	146	190	193	0	0	112	112	0	0	168	170	116	118
52	UR271-XY	XY	238	257	188	192	180	184	146	154	190	193	154	160	112	118	231	231	168	170	116	122
53	UR273-XY	XY	238	257	188	192	180	184	146	154	190	193	156	160	112	118	0	0	168	170	116	122

Table S2: Complete genotype and gender of the 65 unique brown bears of Pindos NP.

A/A	Lab code	Gender	G10H (221-257bp)		Mu26 (182-200bp)		G1D (172-184bp)		G10X (132-154bp)		G1A (180-190bp)		G10P (145-160bp)		G10C (97-126bp)		MU59 (219-251bp)		G10L (153-163bp)		MU50 (110-130bp)	
1	UR315-XY	XY	243	252	192	196	178	180	146	154	190	193	160	160	97	118	239	239	168	170	111	111
2	UR317-XX	XX	243	248	192	196	180	182	146	149	190	193	160	160	97	118	0	0	168	170	109	116
3	UR319-XX	XX	0	0	0	0	184	184	0	0	190	193	0	0	97	118	231	231	168	170	109	116
4	UR320-XY	XY	243	252	196	200	180	182	146	146	185	190	0	0	108	118	251	251	164	168	105	107
5	UR322-XY	XY	234	238	188	192	184	184	0	0	181	181	0	0	112	122	0	0	168	168	107	111
6	UR323-XY	XY	234	238	188	192	184	184	146	146	0	0	160	160	112	122	241	251	160	162	107	111
7	UR324-XX	XX	0	0	196	200	180	184	0	0	190	193	0	0	0	0	251	251	160	160	107	109
8	UR328-XY	XY	234	257	192	200	0	0	146	146	0	0	160	160	112	120	239	251	156	160	107	109
9	UR329-XX	XX	0	0	192	192	184	184	0	0	190	193	156	160	126	126	0	0	168	168	0	0
10	UR331-XX	XX	260	260	192	192	182	184	0	0	0	0	160	160	112	120	0	0	0	0	107	111
11	UR332-XX	XX	238	257	192	196	184	184	146	146	193	193	160	160	122	124	239	239	170	170	109	120
12	UR334-XX	XX	260	260	192	196	0	0	146	146	190	190	150	160	120	124	231	239	170	170	109	111
13	UR335-XY	XY	243	252	192	196	0	0	146	149	190	193	150	156	120	122	239	239	170	170	109	116
14	UR341-XY	XY	234	248	192	192	0	0	146	146	0	0	156	160	97	120	0	0	0	0	105	107
15	UR344-XY	XY	234	243	192	192	0	0	146	154	190	193	160	160	112	122	231	239	160	170	111	120
16	UR347-XY	XY	224	224	192	192	182	182	146	154	193	193	152	160	97	118	239	239	170	170	111	111
17	UR349-XY	XY	234	258	192	196	184	184	146	146	190	193	150	156	120	122	239	241	168	170	107	118
18	UR352-XX	XX	0	0	0	0	184	184	146	146	185	190	0	0	106	106	224	224	168	170	120	120
19	UR353-XY	XY	260	260	0	0	180	182	146	154	193	193	150	156	120	122	239	239	168	170	111	116
20	UR355-XX	XX	260	260	0	0	0	0	0	0	193	193	160	160	97	112	0	0	168	170	109	118
21	UR357-XY	XY	243	252	192	196	0	0	146	146	181	190	150	156	120	124	239	239	170	170	111	118
22	UR358-XY	XY	243	260	192	196	0	0	146	146	190	193	156	160	97	118	0	0	170	170	107	116
23	UR359-XY	XY	243	252	192	196	0	0	146	154	193	193	160	160	97	120	239	251	170	170	109	111
24	UR360-XY	XY	243	252	192	196	0	0	146	154	193	193	160	160	120	124	239	251	168	170	107	111
25	UR361-XY	XY	234	248	192	196	0	0	146	146	190	190	152	160	118	126	0	0	160	160	109	111
26	UR362-XY	XY	243	243	192	196	184	184	146	154	190	193	160	160	97	118	0	0	168	168	111	116
27	UR363-XY	XY	260	260	192	196	0	0	146	146	185	190	160	160	97	122	227	231	168	170	107	109
28	UR365-XY	XY	234	257	192	196	184	186	146	154	190	193	160	160	120	124	231	239	168	170	107	111
29	UR366-XY	XY	234	257	192	196	186	186	146	154	193	193	160	160	97	118	231	241	170	170	111	120
30	UR367-XY	XY	226	234	192	196	0	0	146	154	190	193	160	160	97	122	231	241	168	168	109	116
31	UR370-XY	XY	260	260	200	200	0	0	146	154	190	193	160	160	118	122	251	251	168	170	111	116
32	UR371-XY	XY	260	260	200	200	0	0	146	154	193	193	160	160	118	122	251	251	168	170	111	116
33	UR372-XY	XY	260	260	192	196	0	0	146	154	190	193	160	160	122	124	0	0	170	170	109	111
34	UR373-XX	XX	234	243	0	0	0	0	146	146	190	193	150	152	97	126	0	0	0	0	109	111
35	UR374-XY	XY	243	252	192	196	0	0	146	154	193	193	160	160	97	124	239	239	170	170	111	111
36	UR378-XY	XY	0	0	192	192	0	0	146	146	193	193	0	0	120	124	0	0	164	168	109	116
37	UR379-XY	XY	243	252	192	196	184	184	146	146	185	190	160	160	120	122	239	251	162	162	109	111
38	UR380-XX	XX	260	260	192	192	182	184	154	154	185	190	0	0	97	112	0	0	168	170	107	118
39	UR381-XY	XY	243	252	0	0	0	0	146	154	190	193	0	0	118	122	0	0	156	160	111	116
40	UR382-XY	XY	234	252	192	196	0	0	146	154	190	193	0	0	118	122	239	239	156	160	111	116
41	UR383-XY	XY	234	243	188	192	0	0	146	154	193	193	160	160	97	122	231	239	160	160	111	122
42	UR385-XY	XY	0	0	0	0	0	0	146	146	193	193	160	160	120	120	0	0	168	168	107	109
43	UR392-XY	XY	243	252	0	0	0	0	146	146	185	190	160	160	122	124	0	0	168	170	109	111
44	UR393-XX	XX	243	252	0	0	0	0	146	146	0	0	160	160	124	124	239	239	168	170	109	111
45	UR394-XY	XY	234	243	192	192	0	0	146	146	190	193	160	160	97	118	231	239	170	170	107	107
46	UR395-XY	XY	234	257	192	196	0	0	146	146	190	193	150	160	120	122	231	239	168	170	109	111
47	UR396-XX	XX	260	260	0	0	0	0	146	146	0	0	160	160	118	120	0	0	168	170	111	122
48	UR397-XX	XX	238	238	0	0	184	184	146	146	0	0	0	0	97	118	0	0	170	170	109	111
49	UR398-XY	XY	243	243	192	196	0	0	146	146	190	193	148	150	108	122	0	0	170	170	111	118
50	UR399-XX	XX	0	0	192	192	0	0	0	0	193	193	0	0	100	124	231	231	168	170	109	118
51	UR400-XX	XX	0	0	188	192	182	184	0	0	0	0	156	160	97	110	231	231	0	0	109	111
52	UR402-XX	XX	0	0	192	192	0	0	0	0	190	190	160	160	124	124	0	0	164	168	118	128
53	UR405-XY	XY	224	224	192	196	0	0	146	154	190	193	150	150	97	112	0	0	0	0	111	116
54	UR406-XX	XX	0	0	192	196	184	184	146	154	193	193	0	0	0	0	239	239	0	0	107	107
55	UR409-XY	XY	260	260	192	196	184	184	146	154	0	0	0	0	97	110	0	0	0	0	107	111
56	UR412-XX	XX	0	0	192	196	0	0	141	146	183	190	160	160	97	110	243	243	0	0	107	109
57	UR414-XY	XY	234	248	192	196	0	0	146	146	193	193	160	160	120	124	239	239	170	170	111	120
58	UR416-XY	XY	234	243	192	200	0	0	146	146	190	193	160	160	120	124	241	241	168	170	111	118
59	UR418-XY	XY	234	234	188	196	0	0	146	146	190	193	160	160	97	124	231	239	168	170	109	118
60	UR419-XY	XY	226	226	188	196	0	0	146	154	190	193	160	160	97	120	231	239	170	170	109	116
61	UR422-XY	XY	238	252	188	192	0	0	146	154	190	193	160	160	108	122	0	0	164	168	111	116
62	UR423-XY	XY	226	234	192	196	0	0	146	146	193	193	150	156	120	120	231	251	160	162	107	111
63	UR427-XX	XX	0	0	184	184	0	0	146	146	193	193	0	0	120	122	231	231	0	0	109	111
64	UR428-XY	XY	260	260	192	196	0	0	146	146	190	193	152	160	112	122	231	243	168	170	111	120
65	UR430-XX	XX	243	252	0	0	0	0	146	146	193	193	156	156	118	122	0	0	168	170	111	116

Table S3: Complex genotype and gender of the 77 unique brown bears of Rhodopi NP.

A/A	Lab code	Gender	G10H (221-257bp)		Mu26 (182-200bp)		G1D (172-184bp)		G10X (132-154bp)		G1A (180-190bp)		G10P (145-160bp)		G10C (97-126bp)		MU59 (219-251bp)		G10L (153-163bp)		MU50 (110-130bp)	
1	UR57-XY	XY	257	257	200	200	184	184	0	0	193	193	156	156	108	126	231	239	168	170	122	128
2	UR97-XY	XY	257	257	192	196	182	184	0	0	193	193	150	156	118	120	231	251	168	170	118	118
3	UR101-XX	XX	257	257	0	0	0	0	0	0	177	177	156	156	120	120	231	231	170	170	116	118
4	UR219-XY	XY	257	257	188	188	174	178	146	146	190	190	156	158	110	120	0	0	168	170	116	118
5	UR230-XY	XY	257	257	188	188	0	0	146	154	193	193	156	160	110	118	231	239	168	170	120	120
6	UR102-XY	XY	257	257	188	188	184	184	0	0	177	190	158	160	112	122	231	239	168	170	118	122
7	UR104-XY	XY	257	257	196	200	184	184	0	0	185	190	145	145	97	106	239	251	168	170	118	122
8	UR134-XY	XY	257	257	192	200	184	184	0	0	181	183	145	145	97	108	224	239	168	168	111	111
9	UR136-XY	XY	257	257	188	196	180	180	154	154	190	190	156	160	122	124	231	231	168	170	116	116
10	UR137-XY	XY	257	257	192	196	180	180	154	154	190	190	145	160	122	124	231	243	168	170	116	116
11	UR301-XY	XY	257	257	192	192	184	184	146	154	0	0	0	0	0	0	0	0	168	168	116	120
12	UR306-XY	XY	260	260	200	200	184	184	0	0	0	0	0	0	97	97	0	0	168	168	116	120
13	UR311-XY	XY	257	257	184	200	184	184	141	154	190	193	156	160	110	122	239	239	168	168	116	118
14	UR82-XY	XY	221	221	192	200	0	0	0	0	185	190	145	145	97	110	239	251	168	170	118	122
15	UR91-XY	XY	221	221	192	200	0	0	0	0	193	193	150	158	108	122	239	251	168	170	118	122
16	UR65-XY	XY	221	221	192	200	184	184	0	0	183	185	146	146	97	102	227	231	168	170	120	122
17	UR133-XY	XY	221	221	192	196	184	184	0	0	0	0	145	145	112	114	0	0	168	170	118	120
18	UR74-XY	XY	243	243	192	196	184	184	0	0	185	190	144	146	97	108	239	251	168	170	118	118
19	UR281-XY	XY	232	243	192	200	0	0	0	0	0	0	0	0	97	97	231	231	0	0	116	120
20	UR63-XY	XY	243	243	192	192	184	184	146	146	181	183	156	158	108	108	231	231	168	168	118	118
21	UR73-XY	XY	234	234	192	192	182	182	0	0	177	193	160	160	112	126	239	239	168	170	116	118
22	UR76-XY	XY	0	0	188	192	0	0	0	0	183	185	148	150	97	108	231	239	168	170	116	118
23	UR92-XY	XY	234	234	192	196	182	184	0	0	177	193	158	160	124	126	233	239	168	170	116	118
24	UR222-XY	XY	234	243	192	192	180	180	146	146	193	193	145	154	108	118	0	0	168	170	116	118
25	UR224-XY	XY	234	252	188	192	184	184	146	154	193	193	158	160	122	126	231	231	168	170	116	118
26	UR105-XY	XY	234	234	192	192	182	182	0	0	193	193	158	160	124	126	239	239	168	170	116	118
27	UR130-XY	XY	234	234	192	192	0	0	0	0	193	193	156	160	112	122	0	0	168	168	116	118
28	UR59-XY	XY	234	243	190	192	182	182	146	146	177	190	160	160	126	126	241	241	0	0	116	118
29	UR60-XY	XY	236	266	192	196	184	184	146	146	193	193	156	160	114	120	239	239	168	170	116	120
30	UR278-XY	XY	234	252	188	192	184	184	146	149	0	0	0	0	112	112	0	0	168	170	116	118
31	UR79-XY	XY	243	243	192	192	182	182	0	0	177	190	160	160	112	112	227	227	168	170	111	116
32	UR124-XY	XY	243	243	188	200	0	0	0	0	181	185	148	150	97	97	227	227	168	170	116	118
33	UR126-XY	XY	243	257	188	200	182	182	0	0	181	185	148	150	97	97	231	239	168	170	118	122
34	UR305-XY	XY	238	257	188	196	184	184	154	154	193	193	0	0	124	126	231	239	162	164	118	128
35	UR218-XY	XY	238	252	182	188	180	184	146	154	0	0	156	158	110	120	0	0	168	170	118	120
36	UR110-XY	XY	238	238	192	192	182	182	146	146	183	190	160	160	126	126	239	239	168	170	116	118
37	UR274-XY	XY	238	243	188	192	184	184	146	154	190	193	156	160	112	122	231	231	168	170	103	116
38	UR106-XY	XY	248	257	192	196	184	184	0	0	177	177	158	160	108	124	0	0	170	170	118	120
39	UR120-XY	XY	238	260	196	200	186	186	0	0	181	185	146	150	97	97	239	251	168	170	118	124
40	UR283-XY	XY	248	252	188	192	186	186	146	149	0	0	0	0	0	0	0	0	168	170	118	124
41	UR289-XY	XY	248	257	192	192	186	186	0	0	0	0	0	0	0	0	231	231	168	170	111	116
42	UR299-XY	XY	226	232	188	196	184	184	0	0	190	193	158	160	97	110	0	0	168	168	116	118
43	UR95-XY	XY	252	257	188	192	184	184	0	0	193	193	160	160	120	126	239	239	168	170	116	118
44	UR107-XY	XY	252	257	192	196	184	184	0	0	177	177	160	160	106	122	239	239	168	170	116	118
45	UR303-XY	XY	252	252	188	192	184	184	146	146	181	190	148	156	106	108	231	231	168	170	118	120
46	UR69-XY	XY	0	0	200	200	184	184	146	146	177	190	160	160	126	126	231	231	168	170	118	128
47	UR80-XY	XY	252	252	188	188	182	182	0	0	177	193	156	158	126	126	231	231	168	170	118	124
48	UR66-XY	XY	0	0	188	192	182	184	146	146	177	190	158	158	124	126	231	231	168	170	118	128
49	UR216-XY	XY	0	0	200	200	172	174	149	154	193	193	148	152	108	120	0	0	168	170	111	116
50	UR78-XX	XX	0	0	192	192	0	0	0	0	0	0	150	150	110	126	231	231	168	168	111	116
51	UR89-XX	XX	0	0	196	196	0	0	0	0	190	193	152	156	112	120	239	239	152	152	116	118
52	UR240-XX	XX	0	0	196	200	178	180	0	0	0	0	0	0	112	118	231	231	168	168	116	116
53	UR313-XY	XY	0	0	184	184	184	184	146	154	177	190	158	160	124	126	231	231	168	170	116	128
54	UR209-XY	XY	0	0	192	192	0	0	0	0	190	190	0	0	108	108	231	231	168	168	116	116
55	UR210-XY	XY	0	0	188	188	184	184	146	146	190	193	148	152	110	124	231	231	168	168	116	116
56	UR284-XX	XX	0	0	192	192	0	0	0	0	190	193	0	0	110	110	231	231	168	168	118	120
57	UR286-XY	XY	0	0	200	200	0	0	0	0	183	183	0	0	106	106	231	231	168	168	111	118
58	UR295-XY	XY	0	0	188	192	184	184	154	154	190	190	0	0	0	0	0	0	168	168	116	120
59	UR139-XX	XX	0	0	0	0	178	178	0	0	185	190	0	0	112	118	231	231	168	168	118	122
60	UR234-XY	XY	0	0	192	200	186	186	146	154	185	190	145	145	97	110	0	0	168	170	118	122

Table S4: Complex genotype and gender of the 77 unique brown bears of Rhodopi NP (n=61-77).

61	UR239-XX	XX	260	260	0	0	0	0	0	0	0	0	158	158	100	118	231	231	168	170	118	120
62	UR114-XX	XX	260	260	192	200	184	184	0	0	185	190	160	160	112	112	239	243	168	170	116	120
63	UR116-XY	XY	260	260	188	192	186	186	0	0	181	185	146	150	110	112	231	231	168	170	118	128
64	UR109-XY	XY	260	260	200	200	184	184	0	0	177	190	156	160	122	126	231	251	160	168	116	124
65	UR276-XX	XX	260	260	184	192	0	0	146	154	0	0	0	0	97	97	231	231	0	0	118	120
66	UR312-XY	XY	260	260	184	192	184	184	154	154	190	193	160	160	122	124	231	231	168	170	116	122
67	UR302-XY	XY	260	260	0	0	184	184	149	154	190	193	154	156	106	124	0	0	168	170	118	120
68	UR304-XX	XX	260	260	192	192	0	0	0	0	0	0	0	0	106	106	231	231	0	0	116	122
69	UR279-XX	XX	260	260	192	192	0	0	0	0	0	0	0	0	97	97	231	231	168	168	116	120
70	UR292-XY	XY	260	260	0	0	184	184	0	0	190	190	156	156	112	114	219	219	168	168	116	118
71	UR121-XY	XY	260	260	196	200	186	186	146	154	181	185	148	150	97	97	251	251	168	170	118	122
72	UR131-XY	XY	260	260	192	196	184	184	146	154	177	193	156	160	126	126	231	231	168	170	118	128
73	UR214-XY	XY	260	260	184	200	186	186	154	154	193	193	156	156	106	120	0	0	168	168	111	116
74	UR241-XY	XY	260	260	0	0	184	184	149	154	190	193	156	158	108	122	0	0	168	170	118	122
75	UR81-XY	XY	260	260	188	196	0	0	0	0	177	193	160	160	122	124	231	241	168	170	118	124
76	UR98-XY	XY	260	260	196	200	186	186	0	0	181	183	145	150	97	97	231	231	168	170	118	120
77	UR100-XY	XY	260	260	196	200	186	186	0	0	193	193	158	160	112	120	224	227	160	163	103	105
